
JOURNAL INFORMATION
==============================
NLM Title Abbreviation: Tex Med J (Austin)
NLM Journal ID: 21130590R

Texas Medical Journal

ISSN: 0892-8495
Publisher: Biomedical Journal Digitization Project

ARTICLE INFORMATION
==============================
PMCID: PMC9592012
Article version: 1
Subjects: Books and Magazines

Diseases of the Intestines

Print publication date: 1902 Dec
Volume: 18
Issue: 6
First page: 246
Last page: 246
License: This work is free of known copyright restrictions. For more information, please see PMC Back Issue Digitization.
License URL: https://creativecommons.org/publicdomain/mark/1.0/

==============================

DISEASES OF THE INTESTINES. By Dr. I. Boas, Specialist for Gastro-Intestinal Diseases in Berlin. Authorized translation from the first German edition, with special additions by Seymour Basch, M. D., New York City. With 47 illustrations. Pages, 562; price, $5.00. New York: D. Appleton & Company. 1901.

This work should become very popular in this country, for there is not in America, so far as the writer of this review knows, a detailed and exhaustive treatise on intestinal diseases. Our knowledge of the upper and lower portions of the alimentary tract has been advancing *pasi passu* with other special parts of the body, but that portion of the tract between the stomach and rectum has not been studied to the same practical end.

The present volume embraces much that is new, and the profession generally may be expected to take to it like a novel.

The book is divided into two parts. Part I is devoted to the general division of the subject and embraces (1) the history, (2) the examination of the patient, including the employment of the Röntgen rays in the diagnosis of intestinal diseases, (3) the examination of the feces, (4) the diagnostic value of the examination of stomach contents in intestinal diseases, and (5) the diagnostic value of urinary examination in intestinal diseases. Then follows the general therapeutics of intestinal diseases, including (1) the dietetic treatment of intestinal diseases, (2) hydrotherapeutics, (3) massage and electricity, (4) injections—the enema, intestinal and gastric lavage, gaseous inflations, etc.—and (5) medicinal treatment.

Part II is devoted to the special division of the subject and includes (1) acute and chronic intestinal catarrh, (2) habitual constipation and intestinal displacements, (3) ulcers, round and duodenal, (4) neoplasms, stenosis and obstructions, (5) typhlitis, perityphlitis and appendicitis, with an American *résumé,* including sigmoiditis and pericolitis, (6) diseases of the rectum, and (7) the nervous diseases of the intestines. T. J. B.

---

### Publisher's Department.

### Uterine Disorders.

In quoting from an article of unusual interest which appeared in the *Medical Examiner* by Dr. M. E. Chartier, Faculté de Médecine, Paris, upon the above subject he says: "Among the principal diseases of the uterus which fall under our observation may be mentioned amenorrhea, dysmenorrhea, metritis, endometritis, salpingitis and prolapse uteri.

" 'Neutral reflex' is the main indication or symptom of these diseases, and it can be safely said that an appropriate uterine tonic is the first thing to be resorted to if we want to be successful in our treatment. As a matter of fact, tonics of that class are very numer-
